# A Comprehensive Atlas of Testicular lncRNAs Reveals Dynamic Changes and Regulatory Networks During Sexual Maturation in Tibetan Sheep

**DOI:** 10.3390/ani16020176

**Published:** 2026-01-07

**Authors:** Taotao Li, Huihui Wang, Ruirui Luo, Juanjuan Song, Yi Wu, Meng Jia, Yong Zhang, Youji Ma

**Affiliations:** 1 College of Animal Science and Technology, Gansu Agricultural University, Lanzhou 730070, China; whh20630402@163.com (H.W.); song25286@163.com (J.S.); wy1593050417@126.com (Y.W.); 19560830816@163.com (M.J.); 2 Gansu Key Laboratory of Animal Generational Physiology and Reproductive Regulation, Lanzhou 730070, China; zhangyong@gsau.edu.cn; 3 Animal Husbandry, Pasture and Green Agriculture Institute, Gansu Academy of Agricultural Sciences, Lanzhou 730070, China; luoruirui628@163.com

**Keywords:** Tibetan sheep, testicular development, spermatogenesis, sexual maturation, lncRNA

## Abstract

Tibetan sheep, a dominant livestock species on the Qinghai–Tibet Plateau, is characterized by late sexual maturity and low reproductive efficiency. Although long non-coding RNAs (lncRNAs) are known to play critical regulatory roles in mammalian testicular development and spermatogenesis, their expression dynamics and functions in Tibetan sheep remain poorly understood. In this study, we integrated histological and transcriptomic analyses to profile testicular lncRNAs across three developmental stages: pre-pubertal (3 months), sexually mature (1 year), and adult (3 years). Histological examination showed progressive structural maturation of seminiferous tubules, accompanied by significant increases in testicular weight and serum testosterone levels. RNA sequencing identified 10,857 high-confidence lncRNAs and uncovered extensive reprogramming of the lncRNA transcriptome during sexual maturation, with 7784 lncRNAs differentially expressed between pre-pubertal and post-pubertal stages. Functional enrichment analyses of cis- and antisense-target genes indicated that these lncRNAs were involved in key biological processes, including cell cycle regulation, TGF-β and Hippo signaling pathways, extracellular matrix organization, glycolysis, and apoptosis. Co-expression network analysis further linked upregulated lncRNAs to spermatogenesis-related genes involved in processes such as sperm nuclear condensation (e.g., TNP1) and metabolic support (e.g., PFKP). Our findings demonstrated that lncRNAs coordinate testicular development and spermatogenesis in Tibetan sheep by modulating transcriptional networks, remodeling the cellular microenvironment, and reprogramming energy metabolism. This study provides the first comprehensive atlas of testicular lncRNAs in Tibetan sheep and offers novel insights into the epigenetic regulation of male reproduction in high-altitude mammals.

## Simple Summary

Tibetan sheep are vital to the economy of the high-altitude Qinghai–Tibet Plateau, but their late sexual maturity and low reproductive rates pose a challenge for local herders. To understand this issue, we explored the process of testicular development and sperm production during their maturation. We focused on a class of genetic molecules known as long non-coding RNAs (lncRNAs), which function as master control switches, regulating the activity of other genes. By comparing testes from young, sexually mature, and adult sheep, we discovered that the tissue undergoes major structural transformations, accompanied by dramatic changes in the activity of thousands of lncRNAs during puberty. Our findings show that these lncRNAs help direct sexual maturation by turning key genes on and off. They coordinate essential steps in testicular development, such as cell proliferation, communication between cells, and energy metabolism required for sperm formation. This research provides the first comprehensive map of lncRNAs in the Tibetan sheep testis. It reveals new insights into the molecular mechanisms behind their sexual maturation, offering a foundation for future strategies to improve their reproductive efficiency.

## 1. Introduction

Tibetan sheep (*Ovis aries*), a dominant livestock species on the Qinghai–Tibet Plateau, exhibit a distinctive reproductive profile characterized by late sexual maturity and a relatively low reproductive rate. Specifically, they typically reach sexual maturity around 1 year of age but do not mate until approximately 2.5 years old [1]. Their reproductive process is further characterized by seasonal estrus with an average cycle of 18 days, only one breeding opportunity per year, and a litter size typically limited to a single lamb [2]. These traits represent a major constraint on the sustainability and productivity of high-altitude animal husbandry. Improving flock reproductive performance thus critically depends on enhancing male fertility, for which semen quality serves as a primary indicator. Normal testicular development is fundamental to spermatogenesis, a highly coordinated process involving the precise proliferation and differentiation of germ cells, functional support from somatic cells, and complex regulatory networks of key genes and non-coding RNAs (ncRNAs) [3,4]. Among diverse ncRNAs, long non-coding RNAs (lncRNAs) have attracted considerable attention due to their functional versatility and critical regulatory roles in mammalian biology [5,6,7]. Substantial evidence indicates that lncRNAs are indispensable regulators of key processes in male reproduction, including sex determination [8], hormone secretion [9], and germ cell development [10]. For instance, in mice, the absence of Lnc10 triggers germ cell apoptosis via the p38 MAPK pathway [10], while the spermatogonia-specific lncRNA033862 modulates Gfrα1 expression to maintain the self-renewal and survival of spermatogonial stem cells [11]. Although testis-specific X-linked Tslrn1 knockout does not cause severe infertility, it significantly reduces sperm count [12]. In goats, the nuclear-enriched lncRNA lncNONO-AS influences androgen receptor levels by regulating NONO expression in Sertoli cells [13]. Human studies further associate aberrant expression of specific lncRNAs (e.g., lnc32085, lnc09522, lnc98487) with poor sperm motility, oligospermia, and infertility [14]. Collectively, these findings underscore the central regulatory importance of lncRNAs in mammalian spermatogenesis and male fertility, with roles spanning germ cell fate determination, blood-testis barrier integrity, and modulation of key signaling pathways.

Significant progress has been made in characterizing the stage-specific expression and functions of lncRNAs during testicular development in model organisms such as mice [15] and livestock including pigs [16] and cattle [17]. These studies consistently reveal extensive reprogramming of the lncRNA transcriptome during sexual maturation, highlighting their potential pivotal role in this critical developmental transition. However, systematic investigations of lncRNAs in sheep testicular development and spermatogenesis remain limited, with research on the high-altitude adapted Tibetan sheep being particularly scarce.

Given the established regulatory significance of lncRNAs in male reproduction and the distinct reproductive phenotype of Tibetan sheep, we hypothesize that lncRNAs exert stage-specific regulatory functions during testicular development and functional maturation in this breed. To test this hypothesis, the present study aims to: (1) delineate the dynamic expression landscape of lncRNAs in Tibetan sheep testes across key postnatal developmental stages (pre-pubertal, post-pubertal, and adult); (2) predict their potential cis- and antisense-acting regulatory mechanisms; and (3) construct functional lncRNA-mRNA networks. By integrating morphological and transcriptomic analyses, this research seeks to provide deeper insights into the molecular mechanisms, particularly those mediated by lncRNAs, underlying testicular development and spermatogenesis in Tibetan sheep. The findings may offer a theoretical foundation for understanding their reproductive adaptation and inform potential genetic improvement strategies.

## 2. Materials and Methods

### 2.1. Experimental Animals and Tissue Collection

A total of 24 healthy male Tibetan sheep (half-siblings, sharing the same sire) were obtained from the Ganjia Tibetan Sheep Breeding Cooperative in Gansu Province, China. Animals were divided into three groups representing key postnatal developmental stages: pre-pubertal (3 months, 3 M, *n* = 8), sexually mature (1 year, 1 Y, *n* = 8), and adult (3 years, 3 Y, *n* = 8). All sheep were reared on natural pasture at an altitude of 3000–3500 m under consistent grazing conditions, with ad libitum access to forage and no supplemental feeding. Prior to tissue collection, jugular vein blood samples were collected from each animal for subsequent measurement of serum testosterone concentration using a standard method. The right testis was collected from each animal. To ensure consistency, a transverse section from the mid-region of each testis was uniformly sampled. One portion of tissue was flash-frozen in liquid nitrogen and stored at −80 °C for RNA extraction. Another portion was fixed in 4% paraformaldehyde for 48 h at room temperature, followed by paraffin embedding and hematoxylin and eosin (H&E) staining for histological examination.

### 2.2. RNA Extraction, Quality Control, and Library Construction

Total RNA was extracted from approximately 50 mg of frozen testicular tissue using TRIzol^®^ Reagent (Invitrogen, Carlsbad, CA, USA) according to the manufacturer’s instructions. RNA integrity was assessed by 1.0% agarose gel electrophoresis, and concentration and purity were measured using a NanoDrop spectrophotometer (Thermo Fisher Scientific, Waltham, MA, USA). High RNA quality was confirmed using an Agilent 2100 Bioanalyzer (Agilent Technologies, Santa Clara, CA, USA), with all samples exhibiting an RNA Integrity Number (RIN) > 7.5. Four biological replicates per age group (randomly selected from the eight individuals) were used for transcriptome sequencing. All eight RNA samples per group were reserved for reverse transcription quantitative PCR (RT-qPCR) validation. Strand-specific RNA sequencing (RNA-seq) libraries were constructed using a ribodepletion approach. Ribosomal RNA was removed with the Ribo-Zero™ Gold Kit (Epicentre, Madison, WI, USA). Purified RNA was fragmented, and first-strand cDNA was synthesized using random hexamers and reverse transcriptase. Second-strand cDNA synthesis was followed by purification, end repair, adenylation, and adapter ligation. After size selection and PCR amplification, final libraries were quantified and qualified. Paired-end sequencing was performed on an Illumina HiSeq™ 4000 platform by Gene Denovo Biotechnology Co., Ltd. (Guangzhou, China).

### 2.3. Transcriptome Assembly and LncRNA Identification

Sequencing reads were depleted of ribosomal RNA and aligned to the *Ovis aries* reference genome (Oar_v4.0) using HISAT2 (v2.2.1). Transcript assembly was conducted with Cufflinks, and transcripts from all samples were merged using StringTie in “--merge” mode. Putative lncRNAs were identified via a multi-step filtering pipeline: (1) transcripts shorter than 200 nt or containing fewer than two exons were discarded; (2) transcripts classified by Cuffcompare as “u”, “i”, “j”, “x”, “c”, “e”, or “o” were retained as novel transcripts; (3) coding potential was assessed using CPC and CNCI; and (4) novel transcripts were aligned against the SwissProt database to exclude those with protein homology. The final high-confidence lncRNA set comprised transcripts with no coding potential and no protein annotation.

### 2.4. Differential Expression and Functional Enrichment Analysis

Expression levels of lncRNAs and genes were estimated as FPKM using StringTie [18]. Differential expression analysis between age groups (3 M vs. 1 Y, 1 Y vs. 3 Y, 3 M vs. 3 Y) was performed using the edgeR package (v3.40.2) in R. Significantly differentially expressed (DE) lncRNAs and mRNAs were defined as those with FDR < 0.05 and |log_2_FC| > 1. Cis-regulatory target genes of DE lncRNAs were predicted as protein-coding genes within 100 kb upstream or downstream of the lncRNA locus. Antisense lncRNA–mRNA interactions were predicted using RNAplex [19]. GO and KEGG pathway enrichment analyses for target genes were performed with DAVID and KOBAS, respectively, using a significance threshold of *p* < 0.05.

### 2.5. Co-Expression Network Construction

LncRNA–mRNA co-expression networks were constructed based on FPKM values of DE lncRNAs and their predicted target genes. Pearson correlation coefficients (PCC) were calculated for all lncRNA–mRNA pairs. Pairs with |PCC| > 0.9 and FDR < 0.001 were used to build the network, visualized in Cytoscape (v3.10.3).

### 2.6. RT-qPCR Validation

Ten DE lncRNAs were randomly selected for RT-qPCR validation. Total RNA (500 ng) from each sample was reverse-transcribed using the Reverse Transcription System (Accurate Biotech, Changsha, China). LncRNA-specific primers were designed with NCBI Primer-BLAST (https://www.ncbi.nlm.nih.gov/tools/primer-blast; accessed on 12 April 2025). The primer sequences are listed in Table S1. RT-qPCR was performed in triplicate on a LightCycler^®^ 96 System (Roche, Basel, Switzerland) using TB Green™ Premix Ex Taq™ II (Takara, Kusatsu, Shiga, Japan). Relative expression levels were calculated using the 2^−ΔΔCt^ method, with β-actin as the internal reference.

### 2.7. Statistical Analysis

RT-qPCR data were analyzed using SPSS Statistics (v26.0, IBM, Armonk, NY, USA). Data are presented as mean ± SD. One-way ANOVA followed by Duncan’s test was used for multi-group comparisons. A *p*-value < 0.05 was considered statistically significant (* *p* < 0.05, ** *p* < 0.01).

## 3. Results

### 3.1. Age-Dependent Morphological Changes, Hormone Levels, and LncRNA Characterization in Tibetan Sheep Testes

**Figure 1:**
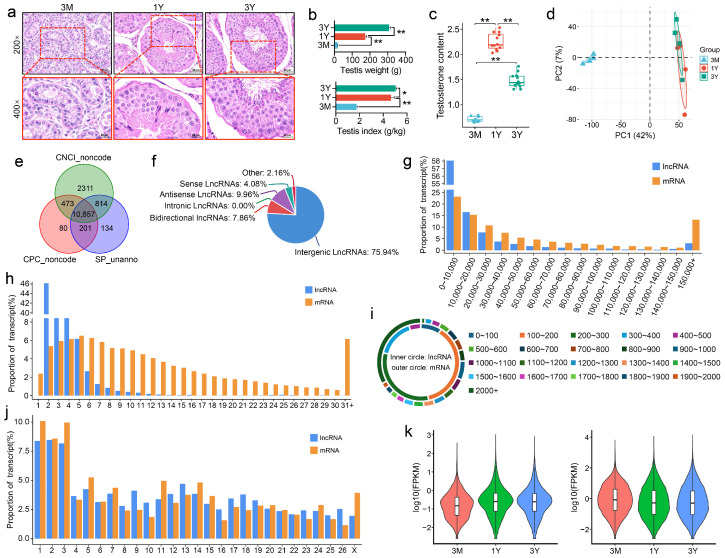
Identification and characterization of lncRNAs in Tibetan sheep testes. (**a**) H&E-stained testicular sections at 3 months (3 M), 1 year (1 Y), and 3 years (3 Y). (**b**) Testicular weight and testis index across age groups. (**c**) Serum testosterone levels. (**d**) Principal component analysis of lncRNA expression profiles. (**e**) Venn diagram of lncRNA identification using CNCI, CPC, and SwissProt. (**f**) Proportional distribution of lncRNA categories. (**g**–**i**) Comparative genomic features of lncRNAs vs. mRNAs: (**g**) transcript length, (**h**) exon number, (**i**) ORF length, (**j**) Chromosomal distribution. (**k**) Expression level comparison. * *p* < 0.05 and ** *p* < 0.01.

H&E staining revealed distinct developmental changes in testicular histology (Figure 1a). At 3 months (3 M, pre-pubertal), seminiferous tubules were small in diameter, lacked a visible lumen, and contained mainly spermatogonia and Sertoli cells, with no post-meiotic germ cells observed; the interstitial tissue ratio was relatively high. At 1 year (1 Y, post-pubertal), tubule diameter increased significantly, the seminiferous epithelium thickened, a clear lumen formed, and a complete spermatogenic sequence from spermatogonia to spermatids was evident. By 3 years (3 Y, adult), tubules further thickened, the lumen was filled with abundant mature sperm, and the epithelium exhibited a more complex and tightly organized structure (Figure 1a). Testicular weight and testis index increased significantly with age (*p* < 0.05, Figure 1b). Serum testosterone levels were significantly higher in the 1 Y and 3 Y groups compared to the 3 M group (*p* < 0.05), but were lower in the 3 Y group than in the 1 Y group (*p* < 0.05, Figure 1c), consistent with age-related physiological changes.

Total RNA from twelve samples was subjected to high-throughput sequencing for lncRNA analysis. An average of 84.13 million raw reads were generated per sample using a 150 bp paired-end strategy. Following quality control, clean reads constituted an average of 98.5% of raw reads per sample. Ribosomal RNA mapping ratios were low (1.02–1.83%). An average of 82.79% (range: 80.38–85.27%) of clean reads aligned to the reference genome. The detailed sequencing metrics are summarized in Table S2. A total of 10,857 lncRNAs were identified through a stringent filtering pipeline integrating CPC, CNCI, and SwissProt databases (Figure 1e). These lncRNAs were classified as: intergenic (75.94%), antisense (9.96%), bidirectional (7.86%), sense (4.08%), and other types (2.16%) (Figure 1f). Compared to mRNAs, lncRNAs were characterized by shorter transcript length (Figure 1g), fewer exons (Figure 1h), and shorter open reading frames (Figure 1i). Chromosomal distribution analysis indicated that both lncRNAs and mRNAs were predominantly enriched on chromosomes 1, 2, and 3 (Figure 1j). Principal component analysis of lncRNA expression showed that 3 M and 1 Y samples were relatively dispersed, whereas 1 Y and 3 Y samples clustered more closely (Figure 1d), reflecting substantial transcriptomic reprogramming during sexual maturation. Overall, lncRNA expression levels were lower than those of mRNAs across all stages (Figure 1k).

### 3.2. Differential Expression Analysis of LncRNAs

**Figure 2:**
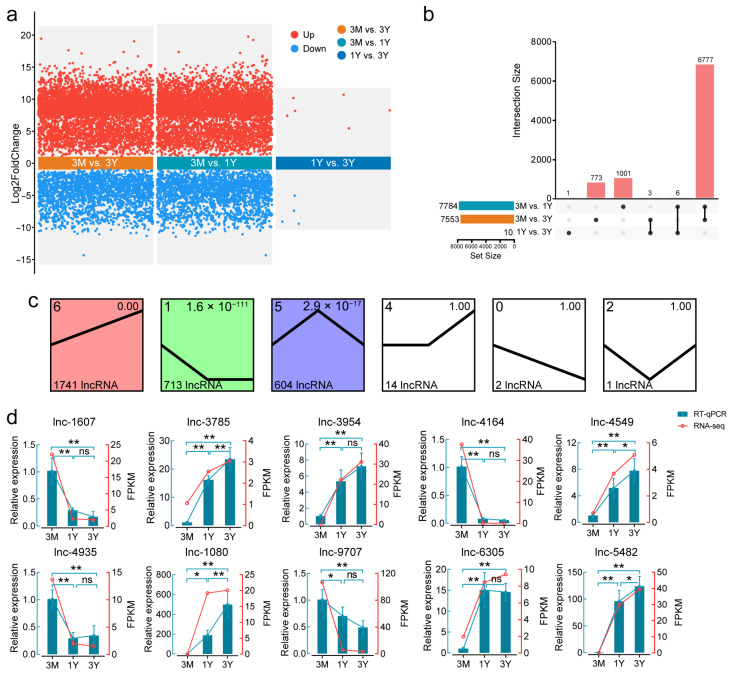
Expression landscape of lncRNA across three developmental stages. (**a**) Volcano plot of DE lncRNAs. (**b**) Upset plot showing numbers of shared and stage-specific DE lncRNAs. (**c**) Six temporal expression patterns identified by cluster analysis. (**d**) Comparison of lncRNA expression trends between RNA-seq and RT-qPCR. * *p* < 0.05, ** *p* < 0.01, and ns, no significant.

A total of 7784 DE lncRNAs were identified between the 3 M and 1 Y groups, with 5402 upregulated and 2382 downregulated (Table S3). In contrast, only 10 DE lncRNAs (6 upregulated, 4 downregulated) were detected between 1 Y and 3 Y (Figure 2a). Venn analysis revealed 1001, 773, and 1 specific DE lncRNAs in the 3 M vs. 1 Y, 3 M vs. 3 Y, and 1 Y vs. 3 Y comparisons, respectively (Figure 2b). The 3 M vs. 1 Y and 1 Y vs. 3 Y comparisons shared 6 lncRNAs, while 3 M vs. 1 Y and 3 M vs. 3 Y shared 6777. DE lncRNAs clustered into six distinct expression patterns (Figure 2c). Clusters 1 (downregulated then stable), 5 (upregulated then downregulated), and 6 (continuously upregulated) were significantly enriched (*p* < 0.05), comprising 713, 1741, and 604 lncRNAs, respectively. RT-qPCR validation of 10 randomly selected lncRNAs confirmed high consistency with RNA-seq trends (Figure 2d), supporting data reliability.

### 3.3. Prediction of Cis-Acting LncRNA Target Genes and Functional Enrichment

**Figure 3:**
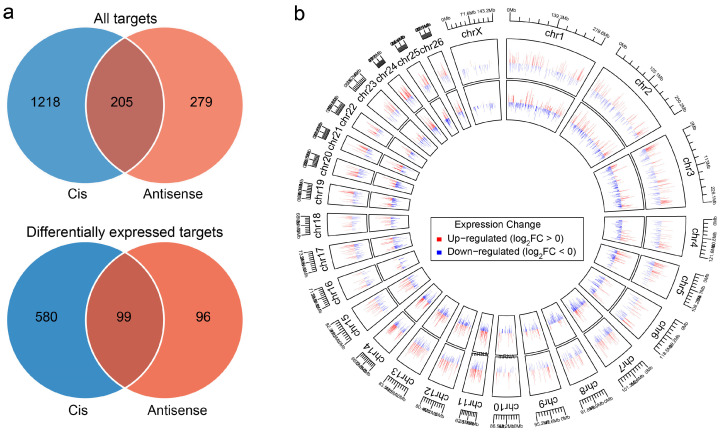
Overlap analysis and chromosomal distribution of cis-lncRNA target genes. (**a**) Venn diagram illustrating the overlap among cis-target, antisense-target, and differentially expressed target genes. (**b**) Circos plot of lncRNAs and cis-target gene distribution across chromosomes.

We identified 1423 cis-target genes and 484 antisense-target genes, with 205 overlapping (Figure 3a; Table S3). LncRNAs and their cis-targets were distributed predominantly on chromosomes 1–3 (Figure 3b). Integration with mRNA expression data yielded 775 differentially expressed target genes (423 upregulated, 714 downregulated; Table S4), including 580 cis-specific, 96 antisense-specific, and 99 common targets (Figure 3b).

**Figure 4:**
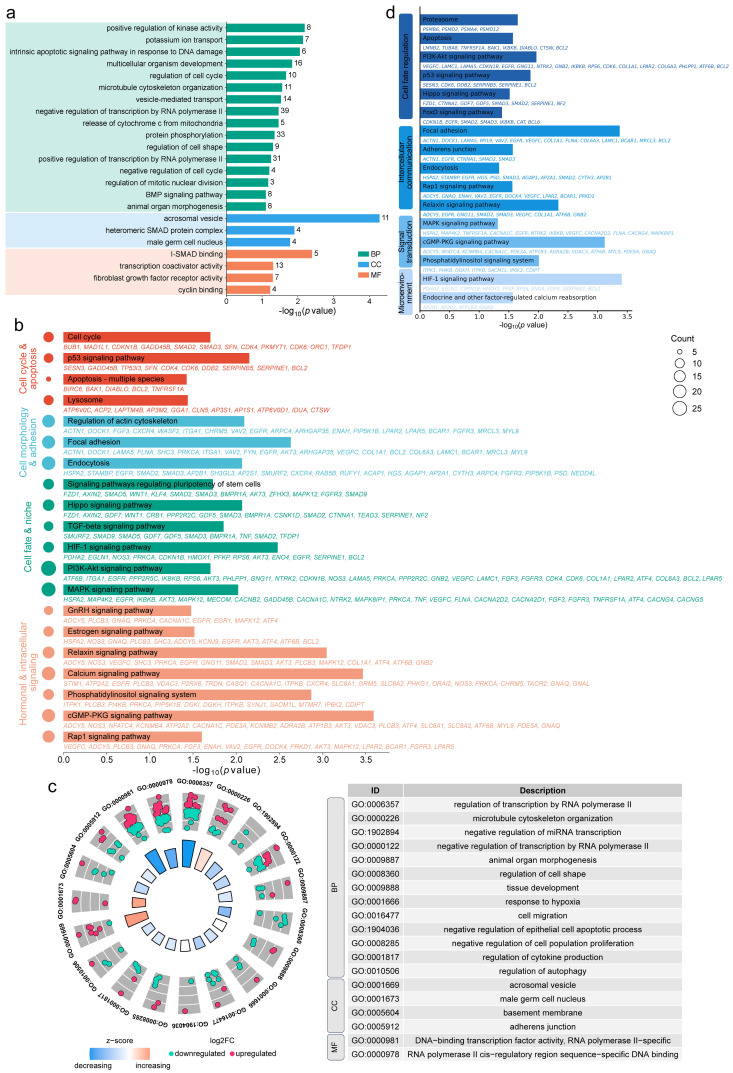
Functional enrichment analysis of cis-acting lncRNA target genes. (**a**,**b**) GO (**a**) and KEGG (**b**) enrichment of cis-target genes of DE lncRNAs. (**c**,**d**) GO (**c**) and KEGG (**d**) enrichment results for differentially expressed cis-target genes.

GO analysis indicated that cis-target genes were significantly enriched in biological processes related to cell division, differentiation, morphogenesis, and apoptosis—such as cell cycle regulation, cell shape modulation, and the BMP signaling pathway. Cellular component terms included spermatogenesis-related structures such as the acrosomal vesicle, receptor SMAD protein complex, and male germ cell nucleus. Molecular function categories were enriched in I-SMAD binding, transcription coactivator activity, and cyclin binding (Figure 4a). KEGG pathway analysis revealed significant enrichment in the cell cycle, p53 signaling pathway, apoptosis, actin cytoskeleton regulation, focal adhesion, TGF-β signaling, Hippo signaling, HIF-1 signaling, stem cell pluripotency regulation, relaxin signaling, estrogen signaling, and GnRH signaling pathways (Figure 4b).

GO analysis of the differentially expressed cis-target genes highlighted enrichment in transcriptional regulation (e.g., RNA polymerase II-mediated transcription, negative regulation of miRNA transcription) and testicular cell organization and morphogenesis (e.g., organ morphogenesis, regulation of cell shape). For cellular components, they were enriched in the acrosomal vesicle, male germ cell nucleus, basement membrane, and adherens junction. Molecular functions were enriched in DNA-binding transcription factor activity and RNA polymerase II cis-regulatory region sequence-specific DNA binding (Figure 4c). KEGG analysis further associated these genes with focal adhesion, adherens junction, apoptosis, proteasome, endocytosis, PI3K-Akt signaling, MAPK signaling, Hippo signaling, FoxO signaling, HIF-1 signaling, Rap1 signaling, and p53 signaling pathways (Figure 4d).

**Figure 5:**
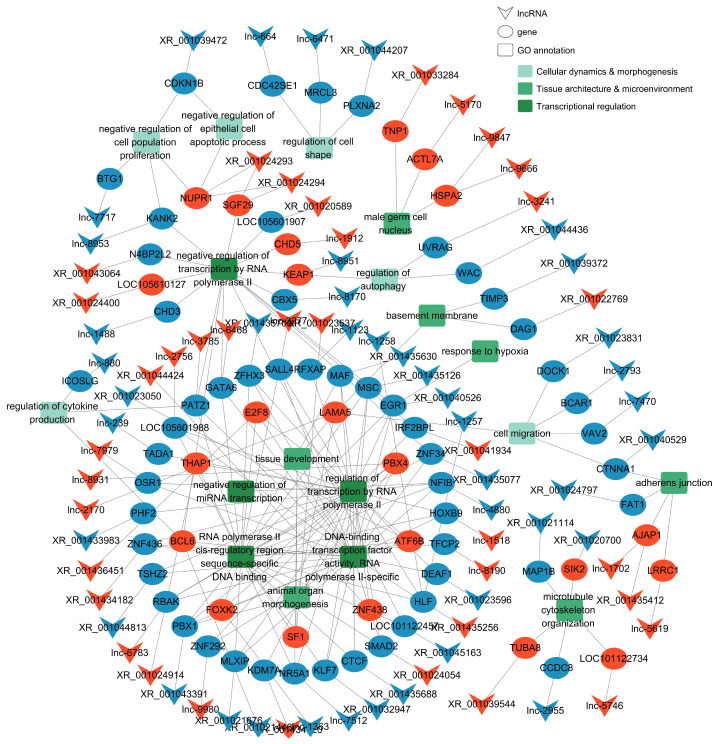
Cis-regulatory lncRNA–mRNA network and representative GO annotations. Red indicates upregulation, and blue indicates downregulation.

Based on cis-acting predictions, a lncRNA-mRNA regulatory network was constructed. After filtering low-abundance transcripts (FPKM < 1.0) and incorporating functional annotations, a final network consisting of 79 lncRNAs (37 upregulated, 42 downregulated) and 75 target genes (22 upregulated, 53 downregulated) was established (Figure 5). This network suggests these lncRNAs may function by targeting key biological processes including transcriptional activity, tissue architecture and microenvironment (e.g., microtubule cytoskeleton organization, male germ cell nucleus, response to hypoxia), and cellular dynamics and morphogenesis (e.g., negative regulation of cell population proliferation, cell migration, regulation of autophagy).

### 3.4. Functional Enrichment Analysis of Antisense LncRNA Target Genes

**Figure 6:**
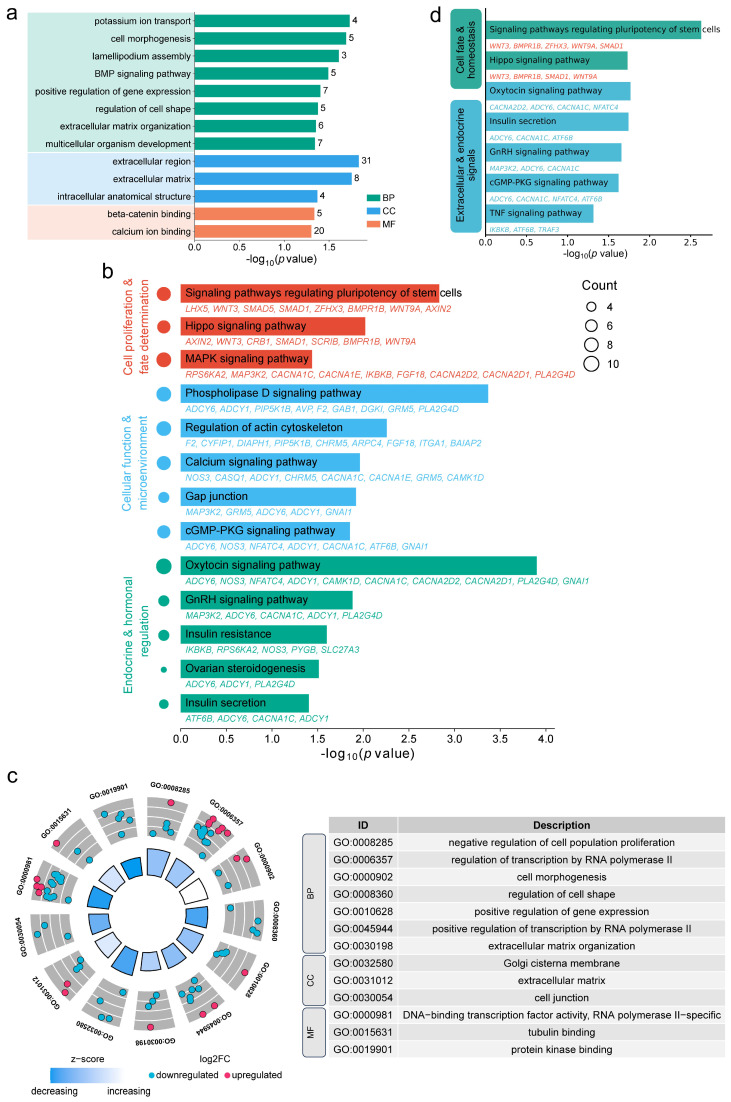
Functional enrichment analysis of antisense lncRNA target genes. (**a**) GO and (**b**) KEGG enrichment for antisense target genes of DE lncRNAs. (**c**) GO and (**d**) KEGG enrichment for DE antisense target genes.

GO analysis of antisense target genes revealed significant enrichment in biological processes including multicellular organism development, cell morphogenesis, the BMP signaling pathway, and positive regulation of gene expression. Cellular component terms included the extracellular region and extracellular matrix. Molecular functions were enriched in beta-catenin binding and calcium ion binding (Figure 6a). KEGG analysis indicated involvement in several spermatogenesis-related pathways, covering endocrine and hormonal regulation (e.g., GnRH signaling, oxytocin signaling, ovarian steroidogenesis, insulin secretion), cell proliferation and differentiation (e.g., Hippo signaling, MAPK signaling, stem cell pluripotency regulation), and maintenance of cellular function and microenvironment (e.g., calcium signaling, cGMP-PKG signaling, gap junction, actin cytoskeleton regulation, phospholipase D signaling) (Figure 6b).

Differentially expressed antisense target genes were primarily enriched in biological processes such as negative regulation of cell proliferation, cell morphogenesis, and extracellular matrix organization. Molecular functions included RNA polymerase II-specific transcription factor activity, tubulin binding, and protein kinase binding. Cellular components were mainly associated with the Golgi membrane, extracellular matrix, and cell junction (Figure 6c). KEGG analysis highlighted pathways regulating stem cell pluripotency, Hippo signaling, oxytocin signaling, GnRH signaling, cGMP-PKG signaling, and TNF signaling (Figure 6d), collectively implicating these genes in germ cell development, hormonal response, and tissue homeostasis during sexual maturation.

**Figure 7:**
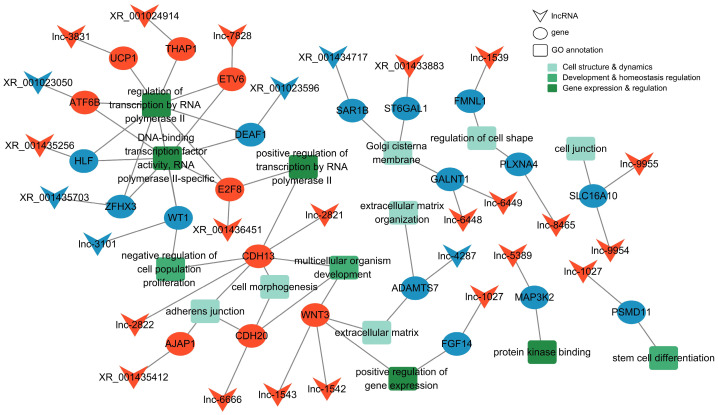
Antisense lncRNA–mRNA network with representative GO annotations. Red indicates upregulation, and blue indicates downregulation.

The antisense lncRNA–mRNA regulatory network comprised 27 lncRNAs (21 upregulated, 6 downregulated) and 23 target genes (9 upregulated, 14 downregulated) (Figure 7), functionally annotated in cell structure and dynamics (e.g., cell morphogenesis, regulation of cell shape, cell junction formation, adherens junction, extracellular matrix composition, extracellular matrix organization), gene expression regulation (e.g., regulation of transcription by RNA polymerase II, positive regulation of transcription by RNA polymerase II, positive regulation of gene expression, DNA-binding transcription factor activity RNA polymerase II-specific, protein kinase binding), and developmental homeostasis (e.g., multicellular organism development, stem cell differentiation, negative regulation of cell population proliferation).

### 3.5. Target Gene Network and Functional Annotation of LncRNAs Upregulated Post-Puberty

**Figure 8:**
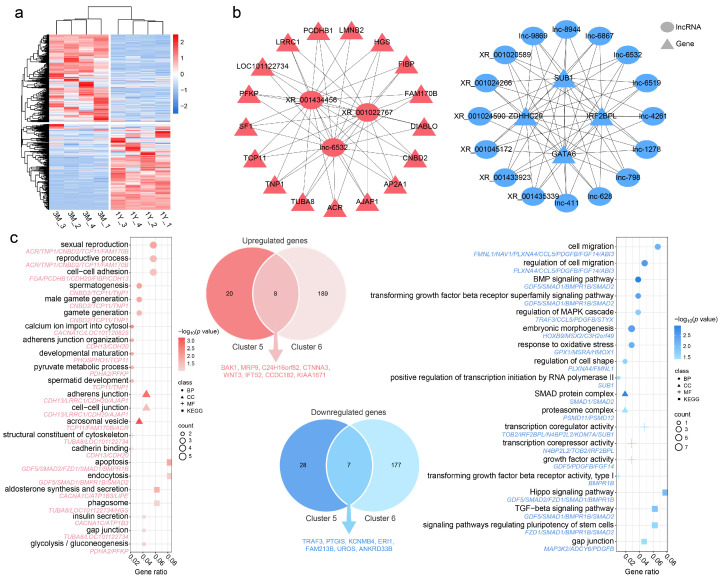
Functional characterization of the target gene network of upregulated lncRNAs during sexual maturation. (**a**) Expression clustering of target genes for lncRNAs differentially upregulated after sexual maturation. (**b**) Co-expression networks of upregulated lncRNAs with positively (**left**) and negatively (**right**) correlated target genes. (**c**) GO (**top**) and KEGG (**bottom**) enrichment for target genes positively (**left**) and negatively (**right**) correlated with upregulated lncRNAs. Red indicates upregulated target genes, while blue indicates downregulated target genes.

From the 648 lncRNAs upregulated post-puberty (clusters 5 and 6), we identified 793 potential target genes, including 429 differentially expressed genes (217 upregulated, 212 downregulated; Figure 8a). GO annotation revealed that upregulated target genes were primarily associated with spermatogenesis, male gamete generation, cell–cell adhesion, adherens junction organization, and other processes related to sperm development and microenvironment homeostasis (Figure 8c). Enrichment of pyruvate metabolic process and structural constituent of cytoskeleton also highlighted the importance of energy metabolism and cytoskeletal remodeling in spermatogenesis (Figure 8c). KEGG analysis further revealed significant activation of the glycolysis/gluconeogenesis pathway, consistent with the energy-supporting role of Sertoli cells. Simultaneously, enrichment in apoptosis and endocytosis pathways reflected post-pubertal tissue remodeling and fine-tuning of testicular function.

Downregulated target genes were mainly enriched in developmental regulation, signal transduction, and stress response (Figure 8c). Specifically, GO terms included cell migration and its regulation, BMP and TGF-beta receptor signaling, and response to oxidative stress, suggesting a transition from developmental processes to functional maturation. KEGG analysis highlighted the Hippo signaling, TGF-beta signaling, and pathways regulating stem cell pluripotency, indicating reduced activity of these pathways after sexual maturity, potentially supporting spermatogenic cell differentiation homeostasis.

Based on expression correlation, we constructed lncRNA–mRNA co-expression networks for both upregulated and downregulated target genes. The top 20 lncRNA–mRNA pairs with the highest correlation (|Pearson correlation| > 0.9, adjusted *p* < 0.001) are shown in Figure 8b. The positive correlation network included 3 lncRNAs (lnc-6532, XR_001434456, XR_001022767) and 17 mRNAs, involving the glycolysis gene PFKP; sperm structural genes TNP1, TCP11, FAM170B, and TUBA8; and spermatogenesis regulators DIABLO, SF1, and ACR. The negative correlation network contained 16 lncRNAs (6 known, 10 novel) and 4 target genes (IRF2BPL, GATA6, SUB1, ZDHHC20), among which GATA6 is implicated in early testicular somatic cell function.

## 4. Discussion

This study delineates the dynamic expression landscape of lncRNAs during testicular development in postnatal Tibetan sheep, integrating histomorphological and transcriptomic analyses from pre-puberty to adult stages. To enhance the detection of developmentally regulated transcripts, we utilized a cohort of half-sibling animals sharing a common sire, a design that reduces inter-individual genetic variance. We note that while this approach strengthens the signal of age-dependent expression changes, it may concurrently introduce paternal genetic influences. Thus, some observed expression differences could reflect a combination of sire-specific effects and physiological maturation. Our findings reveal that testicular maturation involves the structural maturation of seminiferous tubules, germ cell proliferation and differentiation, and fluctuations in serum testosterone, all closely associated with extensive reprogramming of the lncRNA transcriptome. We identified a large set of testicular lncRNAs exhibiting typical features—shorter transcript length, fewer exons, and reduced open reading frame length—consistent with lncRNA characteristics reported in other mammals [20,21], thereby affirming data reliability. These age-dependent lncRNAs are likely key regulators coordinating testicular development, spermatogenesis, and endocrine homeostasis through gene network modulation.

### 4.1. Histological and Endocrine Changes Underline Key Developmental Transitions

The observed histological progression in Tibetan sheep, from seminiferous tubules lacking a lumen and containing only spermatogonia and Sertoli cells at 3 months (pre-pubertal), to expanded tubules exhibiting a complete spermatogenic sequence at 1 year (post-pubertal), and finally to tubules densely packed with spermatozoa in adults (3 years), closely mirrors the classic pattern of testicular maturation in mammals [22,23]. Notably, the tubular morphology in Tibetan sheep at 3 months appears developmentally delayed compared with that of early-maturing, high-fertility breeds. For example, testicular tissue from 2-month-old Small-tailed Han sheep [24], a breed known for early sexual maturity and high fecundity, shows more advanced seminiferous tubule differentiation than is seen in 3-month-old Tibetan sheep. Similarly, when compared with 3-month-old crossbred offspring of Southdown rams and Hu ewes, both recognized for early puberty [23], Tibetan sheep testes display less advanced cytological organization and tubular maturation. These observations align with the late sexual maturity and low reproductive efficiency characteristic of this breed. Concomitant with structural development, serum testosterone concentrations in Tibetan sheep increased significantly from 3 months to 1 year of age, representing a key endocrine driver of pubertal transformation. This rise parallels the marked increase in testosterone observed in human males after puberty onset [25]. As a major androgen, testosterone plays an essential role in promoting the functional maturation of Sertoli cells and the differentiation of spermatogenic cells, thereby ensuring the normal progression of spermatogenesis [25,26].

### 4.2. Dynamic LncRNA Expression Profiles Reflect Key Transitions in Testicular Development

Principal component analysis revealed a pronounced divergence in lncRNA expression between pre- and post-pubertal stages (3 M vs. 1 Y), whereas expression profiles remained relatively stable across post-pubertal time points (1 Y vs. 3 Y). This pattern aligns with the intense tissue remodeling characteristic of puberty, followed by the establishment of homeostatic function in mature testes [27]. Differential expression analysis further supports this trend, in that a substantial number of DE lncRNAs (7784) were identified between 3 M and 1 Y, whereas only 10 were detected between 1 Y and 3 Y. This finding is consistent with reported changes in gene expression numbers across developmental stages in the testes of low-altitude, high-fertility Hu sheep [28]. Moreover, the scale of this change far exceeds the 1118 DE lncRNAs reported during a comparable developmental period (3 M vs. 9 M) in Hu sheep testes [29]. This pronounced difference suggests that sexual maturation in Tibetan sheep involves more extensive or distinct lncRNA-mediated regulation. This may represent an adaptive strategy, where prolonged and finely tuned transcriptional regulation builds a robust reproductive system capable of withstanding persistent plateau pressures. These inferences, however, rely on indirect comparisons. Direct comparative transcriptomics between Tibetan and low-altitude breeds at matched developmental stages are needed to conclusively identify plateau-specific lncRNAs and define their role in high-altitude adaptation. Cluster analysis identified three significantly enriched expression patterns, namely Cluster 1 (downregulated then stable), Cluster 5 (upregulated then downregulated), and Cluster 6 (continuously upregulated). These patterns suggest distinct temporal roles in testicular development and spermatogenesis [3], thereby offering prioritized candidates for functional validation.

### 4.3. Cis-Acting LncRNAs Modulate Core Pathways in Testicular Development and Spermatogenesis

Cis-regulation represents a major mechanism of lncRNA function [30]. Genomically, lncRNAs and their cis-target genes were predominantly enriched on chromosomes 1–3, possibly reflecting both the physical size of these chromosomes and their enrichment for key loci governing testicular and reproductive functions. Through systematic prediction and bioinformatic analysis, we found that cis-target genes were significantly enriched in fundamental processes including cell cycle, differentiation, apoptosis, and BMP signaling—consistent with proteomic changes around sexual maturity reported previously [31]. These processes align with the highly coordinated cellular events during spermatogenesis, such as spermatogonial proliferation, meiosis, and spermiogenesis [32]. Notably, the BMP pathway plays established roles in testicular cord formation and Sertoli cell differentiation during early development, and continues to regulate germ cell dynamics in adults [33]. Differentially expressed cis-target genes were enriched in transcriptional regulation and DNA-binding transcription factor activity, suggesting that lncRNAs may primarily exert regulatory control at the transcriptional level by modulating the function of transcription factors or co-factors [34]. Among these, steroidogenic factor 1 NR5A1 (also known as SF-1) is a core transcriptional regulator critical for the expression of testosterone-producing enzymes, the maintenance of Leydig cell function [35], and the promotion of Sertoli cell maturation [36]. Our cis-acting analysis revealed that NR5A1 was significantly downregulated after sexual maturityand was predicted as a cis-target of the co-downregulated lncRNA lnc-7512. These findings suggest that through its targeting of NR5A1, lnc-7512 may contribute to testicular maturation both by activating steroidogenic enzyme genes for testosterone production and by coordinating Leydig and Sertoli cell development to establish a functional somatic environment prior to sexual maturity.

KEGG analysis further implicated the Hippo, HIF-1, p53, and MAPK pathways, which are key networks governing cell fate, stress response, and microenvironment adaptation [37,38]. The enrichment of pathways such as HIF-1 and MAPK signaling is of particular significance in the context of Tibetan sheep’s high-altitude adaptation. The HIF-1 signaling pathway serves as a central transcriptional regulator in high-altitude mammals, orchestrating cellular responses to hypoxic stress and promoting survival under low oxygen tension [39]. Likewise, a multi-omics study of Tibetan sheep cardiac adaptation to high-altitude hypoxia also identified significant enrichment of the HIF-1 signaling pathway, along with related metabolic and angiogenic processes [40], reinforcing its conserved role in hypoxic adaptation. It has been demonstrated to play a critical role in modulating spermatogenic dynamics and maintaining the homeostasis of the testicular microenvironment [41,42]. Similarly, the MAPK signaling pathway constitutes a key regulatory network enabling cells to respond to various external stimuli, including hypoxic stress [43]. The lncRNAs identified in this study may therefore fine-tune these conserved pathways, potentially contributing to reproductive adaptation to the plateau environment and thereby supporting normal spermatogenesis under hypoxic conditions. The constructed cis-regulatory network, comprising 79 lncRNAs and 75 target genes, offers concrete molecular evidence supporting these inferences. This network suggests these lncRNAs may function primarily through three interconnected aspects: regulating transcriptional activity (directly intervening in gene expression programs), maintaining tissue structure and microenvironment (influencing testis architecture and functional niche), and controlling cellular dynamics and morphogenesis (orchestrating germ cell numbers and morphological development).

### 4.4. Antisense LncRNAs Influence Testicular Maturation via Microenvironment and Transcriptional Regulation

Beyond cis-regulation, we further explored the potential functions of antisense lncRNAs in testicular development during sexual maturation in Tibetan sheep. The results suggest that antisense lncRNAs may play a critical role in testicular tissue microenvironment remodeling and coordination of gene expression programs through a mechanism that is complementary to, yet independent of, cis-acting regulation. Their target genes were notably enriched in extracellular matrix, extracellular region, and cell junctions. Spermatogenesis is a tightly orchestrated process that depends on extracellular matrix degradation and dynamic remodeling of Sertoli cell junctions to enable the directional migration of germ cells across the seminiferous epithelium. The ECM provides structural support and is essential for seminiferous epithelium integrity, Sertoli cell function, and germ cell translocation [44]. Furthermore, the enrichment of target genes in beta-catenin binding and calcium signaling further underscores roles in microenvironmental homeostasis. Beta-catenin regulates cell adhesion and influences spermatogonial state and spermatid differentiation [45,46], while calcium signaling is central to sperm motility and maturation [47]. Collectively, these observations suggest that antisense lncRNAs help establish a stable microenvironment conducive to spermatogenesis by modulating cell adhesion and associated signaling networks.

Analysis of differentially expressed target genes further underscored that involvement of antisense lncRNAs in transcriptional reprogramming and cellular dynamics during testicular maturation. The enrichment of terms related to RNA polymerase II-associated transcription factor activity and positive regulation of transcription implies their direct participation in programmed gene activation during sexual maturation. Within this regulatory landscape, the expression pattern of the transcription factor WT1 is noteworthy. WT1, essential for seminiferous tubule organization, somatic cell (particularly Sertoli cells) development, and spermatogenic homeostasis [48], exhibited high expression prior to sexual maturity, followed by marked downregulation thereafter. This timing is consistent with its known function in developmental remodeling preceding the establishment of mature spermatogenic homeostasis. The lncRNA-mRNA network analysis suggested that the antisense lncRNA lnc-3101, which is downregulated concurrently with WT1, may directly target WT1. These observations suggest that the coordinated downregulation of the lnc-220–WT1 module could contribute to the transcriptional shift from a developmental phase to a mature homeostatic state during testicular maturation, but further functional studies are required to confirm this proposed regulatory mechanism.

Conversely, enrichment associated with negative regulation of cell proliferation and pathways governing stem cell pluripotency suggests these lncRNAs may help maintain spermatogonial stem cell homeostasis, potentially by curbing excessive proliferation to foster directional differentiation. Prior studies have established lncRNAs as master regulators in mesenchymal stem cell homeostasis and multilineage differentiation [49], supporting the notion that they may similarly regulate the stability of the spermatogonial stem cell pool.

### 4.5. Sexual Maturation Reshapes the lncRNA Regulatory Network

Focusing on lncRNAs upregulated post-puberty, we identified 648 candidates and constructed their target gene network. Functional analysis of these targets indicated a shift in testicular priorities from developmental morphogenesis to active spermatogenesis and functional maintenance. Specifically, targets of upregulated lncRNAs were enriched in spermatogenesis, male gamete generation, and energy metabolism pathways such as glycolysis/gluconeogenesis and pyruvate metabolism. Sertoli cells rely on glycolysis to produce lactate, a critical energy substrate for the development and maturation of germ cells [50]. Notably, this finding aligns with observations in Tibetan sheep hearts, where the glycolysis/gluconeogenesis pathway is also reinforced as an adaptation to high-altitude hypoxia [40]. Under hypoxic conditions, efficient anaerobic glycolysis becomes essential for maintaining cellular energy supply [51]. In this context, several upregulated lncRNAs (e.g., lnc-6532, XR_001434456, XR_001022767) showed a positive correlation with PFKP, a key glycolytic enzyme, suggesting their potential role in enhancing glycolytic support from Sertoli cells to sustain spermatogenesis. The upregulation of these PFKP-associated lncRNAs may therefore reflect a metabolic adaptation in Tibetan sheep testes to hypoxia, strengthening glycolysis to meet the energy demands of sperm production.

Additionally, enrichment in cell–cell adhesion and adherens junction organization reflects ongoing structural remodeling of the seminiferous epithelium, which is essential for blood-testis barrier function and germ cell migration [52]. Similar lncRNAs in mice have been shown to regulate germ cell adhesion and junction integrity [53,54]. We also identified lncRNAs correlated with TNP1, a sperm nuclear condensation protein whose dysregulation is linked to impaired chromatin integrity and reduced subfertility [55,56], suggesting that these lncRNAs may fine-tune the expression of key spermatogenic effector genes. Conversely, targets of downregulated lncRNAs were enriched in developmental signaling pathways, such as TGF-β, BMP, and Hippo, which play pivotal roles in embryonic and juvenile testis development [57,58]. Their downregulation after maturity implies a developmental transition from tissue construction to functional maintenance, likely facilitating testicular homeostasis and sustained sperm production.

Although this study provides systematic insights into lncRNA dynamics during testicular maturation in Tibetan sheep, it is important to note that its findings are largely based on computational predictions and correlation analyses. While RT-qPCR confirmed the expression patterns of selected lncRNAs, functional validation remains to be performed. Future studies employing knockdown or overexpression of key candidates, such as those associated with glycolysis (e.g., lnc-6532, XR_001434456) or spermatogenesis (e.g., TNP1-linked lncRNAs), in suitable cellular or organoid models would be valuable to directly test their roles in Sertoli cell metabolism, germ cell differentiation, and testicular microenvironment remodeling.

## 5. Conclusions

This study provides the first comprehensive atlas of lncRNA dynamics in the developing Tibetan sheep testis, highlighting pronounced reprogramming during sexual maturation. Functional analyses demonstrate that these lncRNAs coordinate key biological events in testicular development and spermatogenesis by modulating multiple signaling pathways, including TGF-β, Hippo, and p53, alongside critical processes such as cell junction formation, extracellular matrix organization, and glycolytic metabolism, thereby regulating cellular microenvironment remodeling and energy metabolic reprogramming. These findings offer new molecular insights into male reproductive regulation in high-altitude-adapted mammals and furnish a valuable set of candidate lncRNAs for future functional studies.

## Data Availability

The raw RNA-seq data generated in this study have been deposited in the NCBI Sequence Read Archive (SRA) under the accession numbers SRR11348536 to SRR11348547. These data are publicly accessible at https://www.ncbi.nlm.nih.gov/sra (accessed on 3 November 2024).

## Supplementary Materials

The following supporting information can be downloaded at: https://www.mdpi.com/article/10.3390/ani16020176/s1, Table S1: Primer sequences used for quantitative real-time PCR; Table S2: Overview of lncRNA sequencing data and mapping results. Table S3: Annotation and expression levels of differentially expressed lncRNAs in testes of Tibetan sheep at 3 months and 1 year of age; Table S4: Identification of cis- and antisense-target genes for differentially expressed lncRNAs in Tibetan sheep testes at 3 months and 1 year of age.

## Abbreviations

1 Y	One year of age
3 M	Three months of age
3 Y	Three years of age
ACR	Acrosin
GATA6	GATA-binding protein 6
GO	Gene ontology
H&E	Hematoxylin and eosin
KEGG	Kyoto encyclopedia of gene and genomes
lncRNA	Long non-coding RNA
PCC	Pearson correlation coefficient
PFKP	Platelet-type phosphofructokinase
RNA-seq	RNA sequencing
RT-qPCR	Reverse transcription quantitative PCR
TNP1	Transition nuclear protein 1
TUBA8	Tubulin alpha 8
